# The Diagnostic Accuracy of Transcranial Color-Coded Doppler Ultrasound Technique in Stratifying Intracranial Cerebral Artery Stenoses in Cerebrovascular Disease Patients: A Systematic Review and Meta-Analysis

**DOI:** 10.3390/jcm13051507

**Published:** 2024-03-05

**Authors:** Simon Takadiyi Gunda, Jerica Hiu-Yui Yip, Veronica Tsam-Kit Ng, Ziman Chen, Xinyang Han, Xiangyan Chen, Marco Yiu-Chung Pang, Michael Tin-Cheung Ying

**Affiliations:** 1 Department of Health Technology and Informatics, The Hong Kong Polytechnic University, Hung Hom, Kowloon, Hong Kong SAR, China; simon.gunda@connect.polyu.hk (S.T.G.); jerica.yip@connect.polyu.hk (J.H.-Y.Y.); tsam-kit-veronica.ng@connect.polyu.hk (V.T.-K.N.); ziman.chen@polyu.edu.hk (Z.C.); xinyang-xenia.han@connect.polyu.hk (X.H.); fiona.chen@polyu.edu.hk (X.C.); 2 Department of Rehabilitation Sciences, The Hong Kong Polytechnic University, Hung Hom, Kowloon, Hong Kong SAR, China; marco.pang@polyu.edu.hk

**Keywords:** cerebrovascular disease, stroke, intracranial cerebral arteries stenosis, ultrasonography, transcranial color-coded Doppler, diagnostic accuracy, systematic review and meta-analysis

## Abstract

The early and accurate stratification of intracranial cerebral artery stenosis (ICAS) is critical to inform treatment management and enhance the prognostic outcomes in patients with cerebrovascular disease (CVD). Digital subtraction angiography (DSA) is an invasive and expensive procedure but is the gold standard for the diagnosis of ICAS. Over recent years, transcranial color-coded Doppler ultrasound (TCCD) has been suggested to be a useful imaging method for accurately diagnosing ICAS. However, the diagnostic accuracy of TCCD in stratifying ICASs among patients with CVD remains unclear. Therefore, this systematic review and meta-analysis aimed at evaluating the diagnostic accuracy of TCCD in the stratification of intracranial steno-occlusions among CVD patients. A total of six databases—Embase, CINAHL, Medline, PubMed, Google Scholar, and Web of Science (core collection)—were searched for studies that assessed the diagnostic accuracy of TCCD in stratifying ICASs. The meta-analysis was performed using Meta-DiSc 1.4. The Quality Assessment of Diagnostic Accuracy Studies tool version 2 (QUADAS-2) assessed the risk of bias. Eighteen studies met all of the eligibility criteria. TCCD exhibited a high pooled diagnostic accuracy in stratifying intracranial steno-occlusions in patients presenting with CVD when compared to DSA as a reference standard (sensitivity = 90%; specificity = 87%; AUC = 97%). Additionally, the ultrasound parameters peak systolic velocity (PSV) and mean flow velocity (MFV) yielded a comparable diagnostic accuracy of “AUC = 0.96”. In conclusion, TCCD could be a noble, safe, and accurate alternative imaging technique to DSA that can provide useful diagnostic information in stratifying intracranial steno-occlusions in patients presenting with CVD. TCCD should be considered in clinical cases where access to DSA is limited.

## 1. Introduction

Cerebrovascular disease (CVD) is broadly categorized as a group of conditions affecting blood vessels and blood supply to the brain. In order to harmonize the various classifications and nomenclature of CVD, the term “stroke” is the most appropriately used definition for CVD [1]. According to Feigin et al. [2], stroke is the second leading cause of death and the third leading cause of mortality and disability in the world, whereas ischemic stroke is reported to be the most prevalent subtype of stroke, accounting for more than 80% of all stroke cases [3]. Occlusions or severe stenoses of cerebral arteries (intracranial and extracranial) are associated with plaque build-up within arteries in atherosclerotic disease, and constitute a main parameter for ischemic stroke risk estimation [4]. Carotid artery stenoses account for 10–20% of ischemic strokes [5,6,7,8], whereas in the Asian population, between 33% to 67% of stroke cases are due to intracranial cerebral artery stenoses (ICAS) [9,10]. The early and accurate stratification of ICAS is critical to inform treatment management and enhance prognostic outcomes in CVD patients, as selecting patients to undergo surgical revascularization and thrombolysis is informed by the degree of stenosis [11,12].

Digital subtraction angiography (DSA) is the primary imaging modality for evaluating the cerebral arteries in the diagnosis of atherosclerotic stenosis [13,14]. However, DSA is invasive, expensive, and not readily available. Computed tomography angiography (CTA) and magnetic resonance angiography (MRA) are also used in the assessment of ICAS, but they are expensive and involve the administration of contrast agents. Ultrasonography is a non-invasive, inexpensive, and readily available imaging modality. Duplex carotid ultrasound (DCUS) and transcranial Doppler (TCD) ultrasound are the cornerstone ultrasonography techniques for extracranial and intracranial cerebral arteries assessment, respectively. However, the clinical utility of TCD is limited because it cannot provide anatomical information to allow for precise differentiation between individual vessels, especially in the wake of anatomical variations. Additionally, at a technical level, Doppler angle correction that helps improve the measurement accuracy of blood flow velocities is not possible with TCD. Over recent years, transcranial color-coded Doppler (TCCD) has emerged, and is increasingly becoming available on ultrasound systems and adopted into clinical practice [15]. Contrary to non-imaging TCD, TCCD enables direct visualization of intracranial cerebral arteries through the intact skull by color-coding blood flow velocities [16]. TCCD offers the opportunity for angle correction that enhances the accuracy of blood flow velocity measurements. However, despite the additional technical benefits offered by the TCCD technique, the diagnostic accuracy of TCCD in stratifying ICASs in patients presenting with CVD still remains understudied; hence, further investigation is required. Thus, this study aimed at evaluating the diagnostic accuracy of TCCD in the stratification of intracranial steno-occlusions among patients with CVD, by systematically reviewing and meta-analyzing available literature on the accuracy of TCCD and comparing these results with angiography for the diagnosis of ICAS. It was hypothesized that the TCCD technique has a high diagnostic accuracy in stratifying ICASs.

## 2. Materials and Methods

The study involved a comprehensive search of six electronic databases: Medline, PubMed, Google Scholar, Web of Science (core collection), Cumulative Index to Nursing and Allied Health Literature (CINAHL Complete via EbscoHost), and Embase. The online library of our institution was used to access the databases, and studies published in the English language from 1990 to 5 October 2023 were retrieved. The database search started on 10 February 2023, and the last search was conducted on 5 October 2023. A rerun of the search was conducted prior to the final analysis. The study followed the preferred reporting items for systematic review and meta-analysis (PRISMA) 2020 guidelines, as informed by Page et al. [17].

### 2.1. Search Strategy

The search strategy included searching for concepts related to the PICO framework structured research question, where P (study population) = cerebrovascular disease; I (intervention) = novel ultrasonography imaging technique, transcranial color-coded Doppler ultrasonography; C (comparison) = angiography techniques (DSA, CTA, and MRA), and histopathology used as the reference standards; and O (outcome) = diagnostic accuracy indicators (overall accuracy, sensitivity, specificity, diagnostic odds ratio (DOR), area under the receiver operating curve (AUC)). The concepts were searched using the following: (1) MeSH descriptors in Medline and PubMed, Emtree terms in Embase and CINAHL subject headings, and (2) keywords and their related terms (synonyms, hyponyms). Relevant references from the selected studies were retrieved for further evaluation. The Boolean operator “OR” was used to search within each PICO element concept (MeSH or Emtree terms) and the related entry terms or synonyms, whereas “AND” was applied to search across the concepts of the PICO framework elements. The database search strings are attached in Supplementary Table S1.

### 2.2. Inclusion and Exclusion Criteria

The studies included in this systematic review and meta-analysis were restricted to (1) original studies that were peer-reviewed; (2) those published in the English language; (3) those involving human adults subjects >18 years with suspected cerebrovascular diseases; (4) those assessing the diagnostic accuracy of TCCD technique in the diagnosis of ICAS; (5) those with DSA, CTA, and/or MRA used as the reference standard; (6) those performed in clinical settings; and (7) studies with informed consent and institutional ethical approval obtained prior to data collection.

The exclusion criteria applied to studies that (1) referenced standards other than angiographic imaging techniques (DSA, CTA, MRA) and histopathology; (2) had inadequate information on the diagnostic performance outcome measures; (3) included conference proceedings, posters, case reports, reviews, editorial letters, or commentaries; (4) assessed diagnostic accuracy of non-imaging TCD and other imaging modalities apart from TCCD; (5) were not published in the English language; and (6) involved subjects <18 years who could not give informed consent, and to which CVD is not common.

### 2.3. Data Extraction

The records retrieved from the database were exported to a collaborative systematic review management software—Rayyan (web application, no public version, available at: http://rayyan.qcri.org)—to allow for a collaborative review process. Two reviewers, S.T.G. and T.V.N., independently screened the titles and abstracts of the retrieved records. This was followed by a full text assessment of screened records for eligibility, and a subsequent quality assessment of the eligible studies undertaken by same reviewers. A third reviewer, M.T.C.Y., acted as the moderator, and was responsible for resolving any disagreements between the two independent reviewers. The relevant data pertaining to the authors’ name, date of publication, subjects’ demographic and clinical information, study aim, study methodology, reference standard against which the index test was compared, and the diagnostic performance indicators were extracted from the eligible studies based on the PRISMA 2020 guidelines and recorded on an Excel spreadsheet.

### 2.4. Quality Assessment

The Quality Assessment of Diagnostic Accuracy Studies tool version 2 (QUADAS-2) was utilized to assess the risk of bias and the methodological quality. This involved assessing the risk of bias—applicability concerns in the four domains of (1) patient selection, (2) index test, (3) reference standard, and (4) flow and timing. A total of 14 questions were asked, and each correct answer was awarded a single point according to the methodology by Sultan et al. [18]. The risk of bias and applicability in these domains were categorized into high, low, or unclear, and a meta-analysis was performed in studies that exhibited a minimal risk of bias in the assessed four domains.

### 2.5. Statistical Analysis

Meta-DiSc (version 1.4, the unit of the clinical biostatistics team of Ramón y Cajal Hospital in Madrid, Spain) was used to assess pooled diagnostic performance (sensitivity, specificity, overall accuracy, diagnostic odds ratio, area under the receiver operating curve (AUCROC)) of the eligible studies in the diagnosis and stratification of ICAS. The heterogeneity of the included studies was assessed based on inconsistency (I^2^), and the DerSimonian–Laird random effects model incorporated in Meta-Disc 1.4 was performed to cater to between-study heterogeneity.

## 3. Results

### 3.1. Literature Search

**Figure 1:**
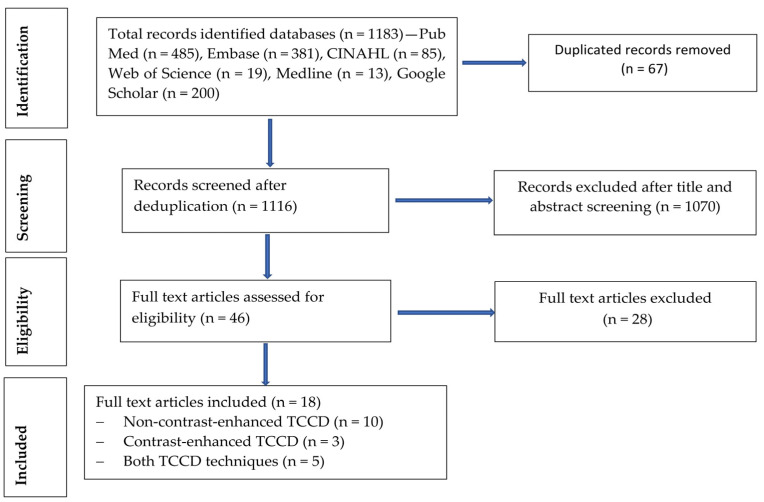
Study selection process (flow chart diagram).

A total of 1183 records were retrieved from the database search, as follows: PubMed (n = 485), Embase (n = 381), CINAHL (n = 85), Medline (n = 13), Web of Science (n = 19), and Google Scholar (n = 200), as shown in Figure 1. The deduplication process identified and removed a total of 67 articles. The remaining 1116 records underwent title and abstract screening, and 1070 articles were excluded. A total of 46 articles were subjected to full text eligibility assessment, and 28 of these articles did not meet the eligibility criteria and were excluded. Finally, a total of eighteen studies met all of the eligibility criteria [19,20,21,22,23,24,25,26,27,28,29,30,31,32]. The main reason for rejection was utilization of imaging modalities other than TCCD such as non-imaging TCD, MRA, and CTA. Additionally, studies that reported reliability indices without assessing the accuracy measures, such as sensitivity, specificity, positive predictive value, negative predictive value, and diagnostic accuracy, were also excluded [33]. Although Gerriets et al. [34] was included in the systematic review, it was excluded from the meta-analysis, as it did not provide adequate measures of diagnostic accuracy. Furthermore, studies that utilized power motion mode TCD (PMD TCD) were excluded [35,36]. The study by Liu et al. [37] was excluded, as no full article could be retrieved, regardless of the study reporting the diagnostic accuracy of TCCD with DSA as the reference standard.

### 3.2. Study Characteristics

The eighteen studies comprised fourteen prospective studies [19,20,22,24,26,27,28,29,30,31,32,38,39,40] and four retrospective studies [21,23,25,41] (Table 1 and Table 2). The total number of patients in the included studies who underwent both index and reference tests was 3082, consisting of 2092 males (68%) and 990 females (32%). A total of 13 studies reported the trans-temporal window (TTW) failure rate, and the pooled mean TTW failure rate was 13.5% (Table 3). The period between TCCD and reference standard imaging examination of the included studies varied, ranging from as little as 12 min to as much as within 90 days (Table 2).

The included studies were observed to exhibit significant study methodological variability with respect to the (1) TCCD techniques—contrast-enhanced versus non-contrast TCCD, (2) ultrasound diagnostic parameter, (3) assessment site, (4) degree of stenosis at which accuracy was established, and (5) reference standard used (DSA, MRA, or CTA). A total of seven (39%) studies [24,26,28,29,38,39,40] used contrast-enhanced TCCD technique alone whereas nine (50%) studies [19,21,23,25,27,30,31,32,41] used solely non-contrast TCCD technique. A combination of the two techniques was utilized in two studies (11%) [20,22] (Table 3). Our study further observed variations in the ultrasound contrast agents used among the included studies, with Levovist (Schering AG, Berlin Germany) and SonoVue (Branco, Italy) being the commonly used contrast agents. No complications or serious side effects associated with the use of contrast agents were reported in the studies. Additionally, we observed that different types of ultrasound machines were used across the studies, but they all used a phased array transducer with a frequency range of 1–5 MHz for the TCCD examinations (Table 2).

With respect to the site of assessment, a majority of the studies assessed the anterior circulation with a focus on middle cerebral artery (MCA) stenoses—nine (50%) studies [22,29,30,31,32,38,39,40,41]. The other seven (39%) studies assessed both anterior and posterior circulations [19,20,21,23,24,27,28], whereas two (11%) studies focused on posterior circulation, mainly the basilar artery (BA) [25,26].

Peak systolic velocity (PSV) and mean flow velocity (MFV) represented the main TCCD ultrasound diagnostic parameters for stenosis assessment. PSV-based diagnosis of ICAS was reported in 14 out of 30 (47%) analyses of the 18 studies, and MFV in 8 (27%) (Table 3). However, the cutoff values of these two ultrasound diagnostic parameters varied across the different degrees of stenosis and sites of assessment. The PSV cut-off values for the diagnosis of greater than or equal to 50% stenosis in the MCA, excluding specific categories (70–99%), ranged from 140 to 220 cm/s with a pooled mean of 186 ± 34 cm/s, whereas the MFV ranged from 80 to 110 cm/s with a pooled mean of 95 ± 21 cm/s. Additionally, for BA stenoses greater than or equal to 50%, the PSV cut-off values ranged from 120 to 150 cm/s with a pooled mean of 137 ± 15 cm/s, and the corresponding MFV ranged from 65 to 90 cm/s with a pooled mean of 78 ± 18 cm/s. The diagnostic criteria for totally occluded MCAs were mainly absent flow signals in the observed vessel, whilst Doppler signals were observed in anterior cerebral artery (ACA) or contralateral MCA.

### 3.3. Studies’ Methodological Quality Assessment Based on QUADAS 2 Tool

The results on the methodological quality of the included studies based on QUADAS version 2 are shown in Table 4. There was generally low risk of bias and applicability concerns across the domains of patient selection, index test, reference standard, and flow and timing in the eligible studies.

### 3.4. Diagnostic Accuracy of TCCD Technique for the Detection of Steno-Occlusive Disease

#### 3.4.1. (DSA, MRA, CTA) as Reference Standards

**Figure 2:**
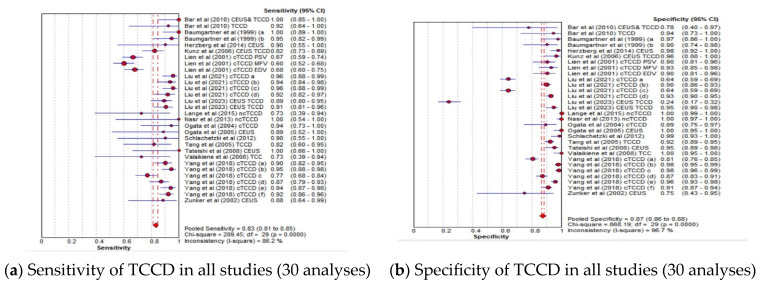

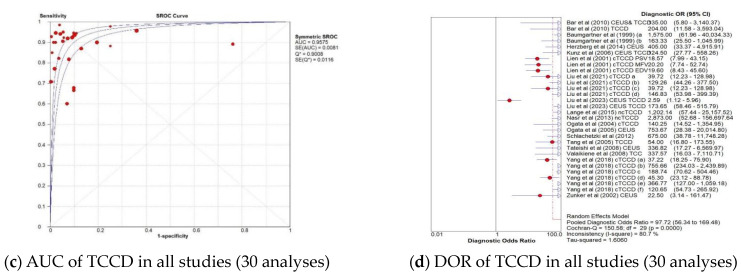
Diagnostic accuracy indicators of TCCD technique when all studies (30 analyses) using either DSA, MRA, CTA, or any combination of the three imaging modalities as reference standards are considered. (**a**) Sensitivity of TCCD in all studies (30 analyses), (**b**) Specificity of TCCD in all studies (30 analyses), (**c**) AUC of TCCD in all studies (30 analyses), (**d**) DOR of TCCD in all studies (30 analyses). The position of the red circles corresponds to the diagnostic accuracy indicator value for each individual study, whilst the position of the red diamond shaped box represents the pooled diagnostic accuracy indicator value.

The pooled diagnostic accuracy indicators of the TCCD technique for 30 analyses utilizing either DSA, MRA, CTA, or a combination of these three imaging modalities as reference standards are shown in Figure 2a–d. The pooled sensitivity, specificity, AUC, and DOR were 83% (95% CI: 81–85%), 87% (95% CI: 86–88%), 0.96, and 98 (95% CI: 56–169), respectively.

#### 3.4.2. TCCD Compared to Only DSA as Reference Standard

**Figure 3:**
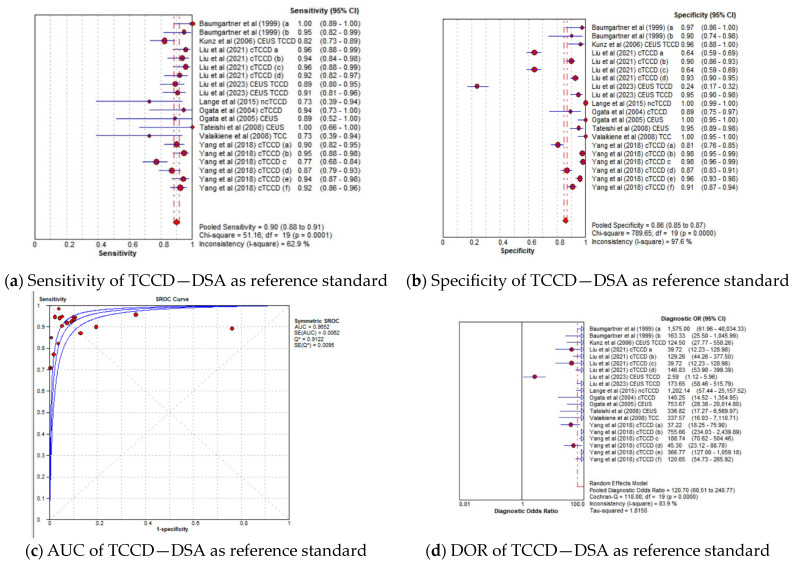
Diagnostic accuracy indicators of TCCD technique when compared to only DSA as the reference standard. (**a**) Sensitivity, (**b**) Specificity, (**c**) AUC, (**d**) DOR. The position of the red circles corresponds to the diagnostic accuracy indicator value for each individual study, whilst the position of the red diamond shaped box represents the pooled diagnostic accuracy indicator value.

The pooled diagnostic accuracy results for the subgroup analysis in which TCCD was compared to solely DSA as the reference standard are shown in Figure 3a–d. TCCD demonstrated good diagnostic performance and the pooled sensitivity, specificity, AUC, and DOR were 90% (95% CI: 88–91%), 86% (95% CI: 85–87%), 0.97, and 121 (95% CI: 61–169), respectively.

#### 3.4.3. Diagnostic Accuracy of TCCD Technique According to Stenosis Categories

**Figure 4:**
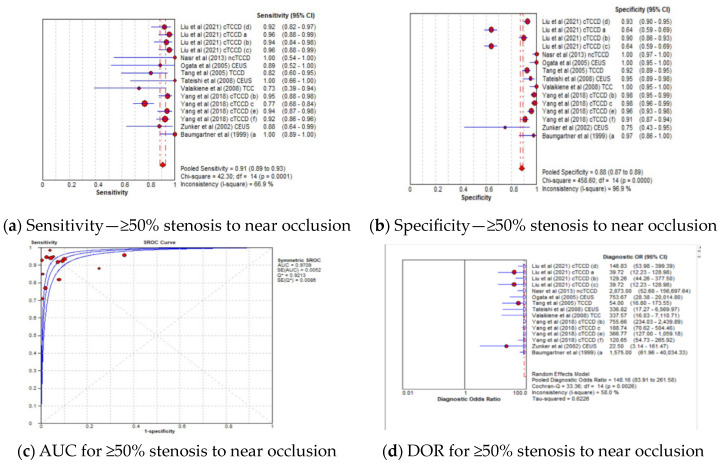
Diagnostic Accuracy of TCCD Technique for stratifying ≥50% Stenosis to Near Occlusion stenosis. (**a**) Sensitivity—≥50% stenosis to near occlusion, (**b**) Specificity—≥50% stenosis to near occlusion, (**c**) AUC for ≥50% stenosis to near occlusion, (**d**) DOR for ≥50% stenosis to near occlusion. The position of the red circles corresponds to the diagnostic accuracy indicator value for each individual study, whilst the position of the red diamond shaped box represents the pooled diagnostic accuracy indicator value.

**Figure 5:**
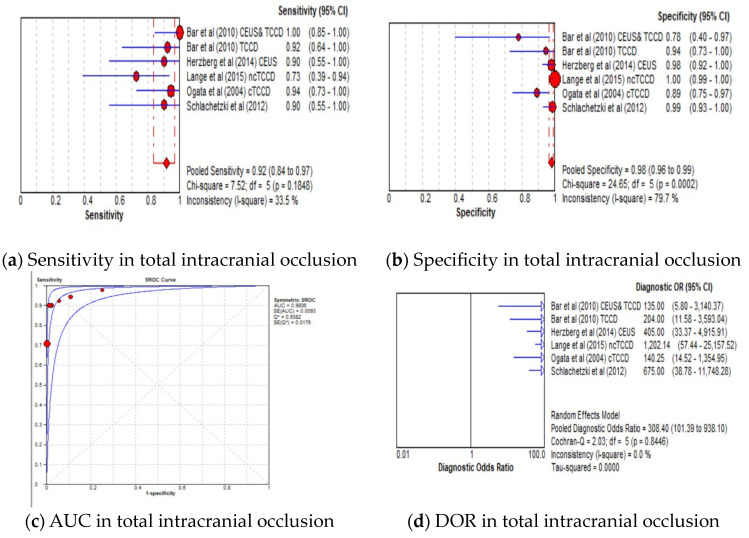
Diagnostic Accuracy of TCCD Technique for the diagnosis of total intracranial occlusion. (**a**) Sensitivity—total intracranial occlusion, (**b**) Specificity—total intracranial occlusion, (**c**) AUC—total intracranial occlusion, (**d**) DOR—total intracranial occlusion. The position of the red circles corresponds to the diagnostic accuracy indicator value for each individual study, whilst the position of the red diamond shaped box represents the pooled diagnostic accuracy indicator value.

The pooled sensitivity, specificity, AUC, and DOR of the TCCD technique in stratifying stenoses ≥50% to near occlusion were 91% (95% CI: 89–93%), 88% (95% CI: 87–89%), 0.97, and 148 (95% CI: 84–262), respectively (Figure 4a–d). The corresponding pooled sensitivity, specificity, AUC, and DOR for diagnosing total occlusions were 92% (95% CI: 84–97%), 98% (95% CI: 96–99%), 0.98, and 148 (95% CI: 84–262), respectively (Figure 5a–d). Although the TCCD technique yielded a good and comparable diagnostic accuracy with angiographic techniques (DSA, MRA, and CTA) in stratifying both stenoses greater than or equal to 50% to near occlusion and total occlusion, the diagnosis of total occlusion by TCCD had a higher specificity in the diagnosis of total occlusion (98%) when compared to the diagnosis of stenosis ≥50% to near occlusions (88%). The observed high specificity could probably be explained by the fact that it is easier to exclude the possibility of total occlusion when color or spectral Doppler signals are observed.

#### 3.4.4. Diagnostic Accuracy of TCCD Technique According to Ultrasound Parameters

**Figure 6:**
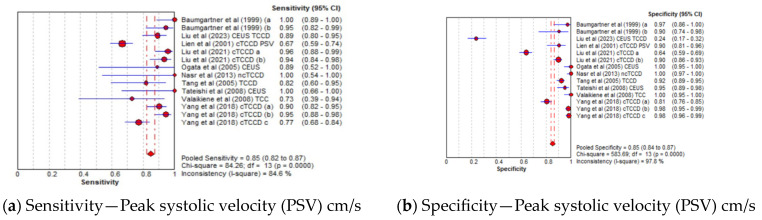

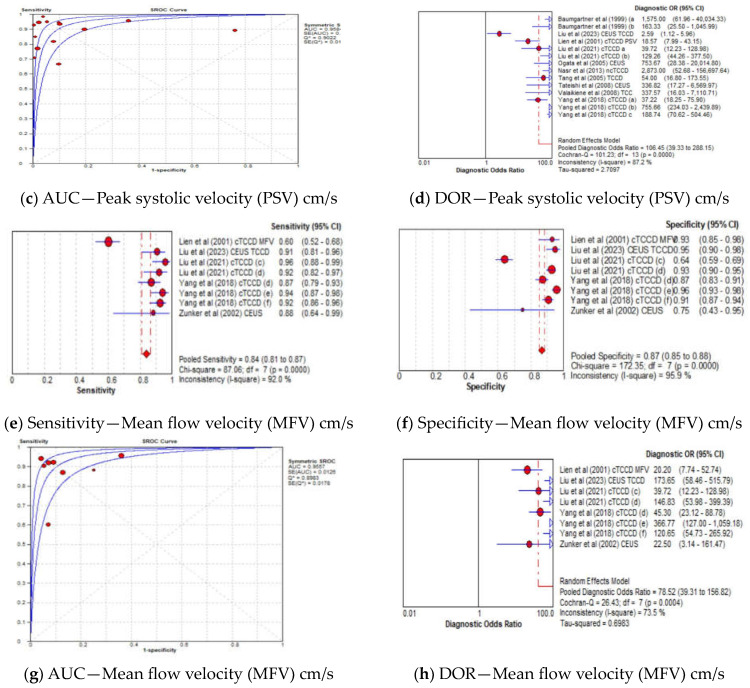
Diagnostic Accuracy of TCCD technique for the two ultrasound parameters—Peak systolic velocity and Mean flow velocity. (**a**) Sensitivity—Peak systolic velocity (PSV) cm/s, (**b**) Specificity—Peak systolic velocity (PSV) cm/s, (**c**) AUC—Peak systolic velocity (PSV) cm/s, (**d**) DOR—Peak systolic velocity (PSV) cm/s, (**e**) Sensitivity—Mean flow velocity (MFV) cm/s, (**f**) Specificity—Mean flow velocity (MFV) cm/s, (**g**) AUC—Mean flow velocity (MFV) cm/s, (**h**) DOR—Mean flow velocity (MFV) cm/s. The position of the red circles corresponds to the diagnostic accuracy indicator value for each individual study, whilst the position of the red diamond shaped box represents the pooled diagnostic accuracy indicator value.

The pooled sensitivity, specificity, AUC, and DOR of utilizing PSV as the diagnostic parameter were 85% (95% CI: 82–87%), 85% (95% CI: 84–87%), 0.96, and 106 (95% CI: 39–288), respectively (Figure 6a–d), whereas the pooled sensitivity, specificity, AUC, and DOR of using MFV as the diagnostic parameter were 84% (95% CI: 81–87%), 87% (95% CI: 85–88%), 0.96, and 79 (95% CI: 39–157), respectively (Figure 6e–h). The cut-off values of the TCCD ultrasound parameters varied for each stenosis category and assessment site, as shown in Table 3. Since both TCCD diagnostic parameters (PSV and MFV) were observed to yield high and comparable diagnostic performance with similar AUC (96%), the two parameters were considered useful in stratifying ICAS among CVD patients.

#### 3.4.5. Diagnostic Accuracy of TCCD Based on Contrast-Enhanced and Non-Contrast TCCD

**Figure 7:**
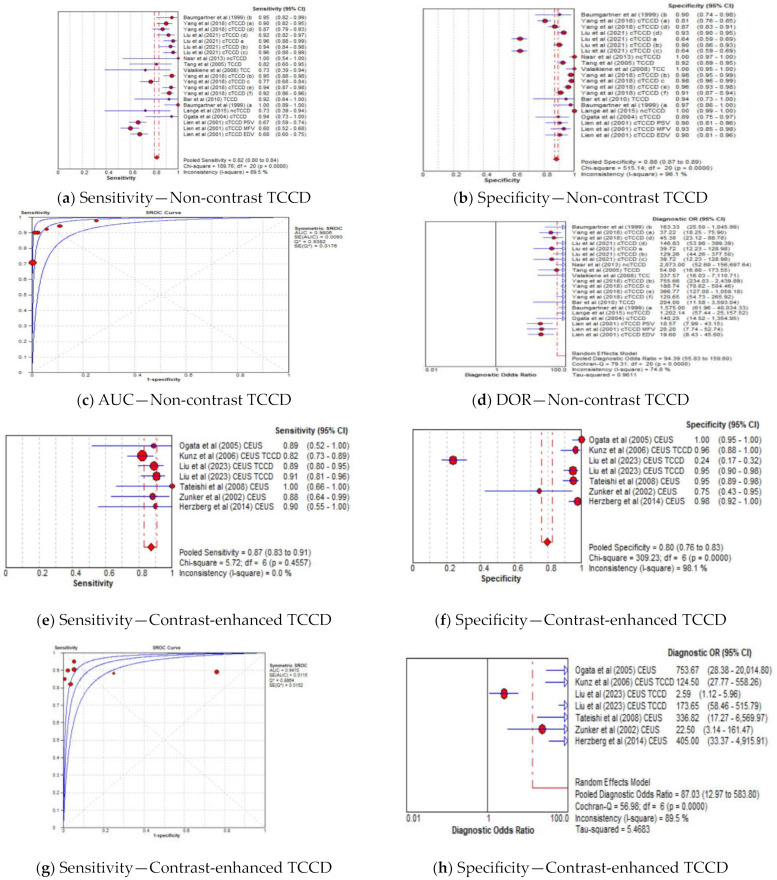
Diagnostic Accuracy of TCCD for the two techniques of contrast-enhanced and non-contrast TCCD. (**a**) Sensitivity—Non-contrast TCCD, (**b**) Specificity—Non-contrast TCCD, (**c**) AUC—Non-contrast TCCD, (**d**) DOR—Non-contrast TCCD, (**e**) Sensitivity—Contrast-enhanced TCCD, (**f**) Specificity—Contrast-enhanced TCCD, (**g**) Sensitivity—Contrast-enhanced TCCD, (**h**) Specificity—Contrast-enhanced TCCD. The position of the red circles corresponds to the diagnostic accuracy indicator value for each individual study, whilst the position of the red diamond shaped box represents the pooled diagnostic accuracy indicator value.

The pooled sensitivity, specificity, AUC, and DOR of utilizing non-contrast TCCD were 82% (95% CI: 80–84%), 88% (95% CI: 87–89%), 0.98, and 94 (95% CI: 56–160), respectively (Figure 7a–d), whereas the pooled sensitivity, specificity, AUC, and DOR of using contrast-enhanced TCCD were 87% (95% CI: 83–91%), 80% (95% CI: 76–83%), 0.95, and 87 (95% CI: 13–584), respectively (Figure 7e–h). The use of contrast enhancement did not improve the overall diagnostic accuracy of TCCD, although the pooled sensitivity was observed to improve (from 82% to 87%) with the use of contrast enhancement.

The results showing the pooled diagnostic performance of TCCD in stratifying ICAs according to various categories are summarized in Table 5.

## 4. Discussion

Although DSA still remains the gold standard for stratification of intracranial steno-occlusions, its limitations such as increased cost, invasiveness nature, and associated complications cannot be overemphasized [43,44], hence the need to investigate alternative imaging techniques. In the present study, we provided evidence pertaining to the diagnostic accuracy of the TCCD technique, which is non-invasive and inexpensive in stratifying ICASs in patients presenting with CVD.

The QUADAS assessment concluded there is high methodological quality within individual studies, as most of the studies had a low risk of bias across the four domains of patient selection, index test, reference standard, and flow and timing (Table 4). A majority of the studies (78%) adopted a prospective study design [19,20,22,24,26,27,28,29,30,31,32,38,39,40], whilst retrospective study design was used in only four studies (22%) [21,23,25,41]; this ensured a low risk of patient selection bias. Additionally, a single-center approach was adopted in all, except one study by Gerriets et al. [40], in which a multicenter approach was used, and this further reduced the possibility of bias and traps related to a multicenter study design [45]. Furthermore, a consecutive participants selection strategy was employed in all except for three studies [23,24,39], which further strengthened the methodological quality and consistency in the domain of patient selection among the included studies. We further observed a significant percentage in gender difference in the patients who underwent both the index and reference tests, (males = 68% versus females = 32%). The observed discrepancy could probably be attributed to previously reported differences in the prevalence rates of cerebrovascular disease across gender groups. Although temporal window failure was reported in previous studies to be higher in the female population compared to male counterparts, no formal analysis on temporal window status across gender was conducted in the current study due to limited information provided in the included studies.

The reference standard tests were clearly specified and described to allow for replication, as well as undertaken by teams, blinded to results of TCCD examination in all included studies. However, some studies utilized more than one reference standard for confirming the diagnosis [22,29,40]. Gerriets et al. [34] utilized three different reference standards (CTA, MRA, and DSA), whereas CTA and MRA were used in Schlachetzki et al. [22] and Herzberg et al. [29]. The use of multiple reference standards in a single study could introduce some applicability concerns with respect to the consistency in the reference index domain, as the accuracy of the three tests is reported to be different.

Notwithstanding, a low risk of bias observed within individual studies, a significant methodological variability across the studies with respect to mainly the (1) TCCD technique-with or without contrast enhancement, (2) ultrasound diagnostic parameter, (3) site assessed (4) degree of stenosis at which the accuracy was established, and (5) reference standard used (DSA, MRA, CTA) was noted. We observed a statistically significant heterogeneity within the study results when all studies were included (Figure 2a–d). The inconsistency index of ≥50% and *p* < 0.001 were deemed to indicate substantial between-study heterogeneity, as alluded to by Castaldo et al. [46]. This observation prompted the undertaking of various subgroup analyses. Although different subtypes of CVD such as AIS and TIA were reported in the included studies (Table 2), our study did not perform a subgroup analysis on different types of stroke, as it has been reported that there is no significant difference in the Doppler ultrasound diagnostic parameters among different types of stroke [38].

In the current meta-analysis, TCCD was observed to yield high diagnostic accuracy in the stratification of ICAS among patients with CVD, when compared to DSA as a reference standard, with a pooled sensitivity, specificity, AUC, and DOR of 90%, 86%, 0.97, and 121, respectively (Figure 3a–d). When studies utilizing DSA, MRA, CTA, or a combination of the three modalities as reference standards were considered, the pooled sensitivity decreased considerably from 90% to 83%, although the overall diagnostic accuracy between the two subgroups remained comparable (97% versus 96%, respectively). These results suggest that the accuracy of TCCD in the stratification of ICAS in patients with CVD is comparable to DSA; TCCD is non-invasive, relatively inexpensive, more acceptable to patients, and has a lower risk of post-examination complications when compared to DSA. We further found that both PSV and MFV are useful TCCD diagnostic parameters for stratifying ICASs, as they yield a comparable and high diagnostic accuracy (both AUC = 0.96) (Figure 6c,g). The diagnosis of ≥50% stenosis has clinical significance for timely treatment and intensive follow-up in our study; hence, pooled mean ultrasound parameter (PSV and MFV) cut-off values for ≥50% stenosis are reported to inform clinical decision making.

Although contrast-enhanced TCCD increased the visualization of intracranial vessels and had higher sensitivity compared to non-contrast TCCD (87% versus 82%, respectively) [22,24,39,40], our meta-analysis demonstrated that contrast enhancement did not result in improved overall diagnostic accuracy in stratifying ICASs (AUC = 0.95 versus 0.98, respectively).

There are limitations in this study. There were only a few studies that met the inclusion criteria, and this could restrict the generalizability of the study results. Notwithstanding the contributions of ICAS to ischemic stroke, recent evidence is pointing towards vulnerable atherosclerotic plaque rupture as a main mechanism of ischemic stroke [14,47]. There is need for future studies to interrogate the diagnostic accuracy of novel imaging techniques that may allow for the early and accurate characterization of plaque morphology, to which our current study was limited.

## 5. Conclusions

In conclusion, this systematic review and meta-analysis provide evidence that the TCCD imaging technique exhibits high diagnostic performance in the stratification of intracranial steno-occlusions among patients presenting with CVD, when compared to DSA as a reference standard. TCCD has potential to be used in stratifying ICASs in CVD patients, and could be considered in clinical cases where DSA is limited or contraindicated to patients.

## Figures and Tables

**Table 1:** Main characteristics of patients in the included studies.

Author (s), Publication Year	Ref.	Country	Type of Patient				n—Number of Patients; * n—Number of Stenosis/Occlusions ** n—Number of Datasets According to Reference Standard, *** n—Number of Vessels							Mean Age ± SD (Years)	Gender	
				Total (n)	Index + Ref Tests (n)	Accuracy Analysis	No Stenosis	<50% Stenosis	≥50% Stenosis Near Occlusion	50−69%	70−99%	Total Occlusion	≥50% Stenosis + Total Occlusion		Male (n)	Female (n)
Baumgartner et al. (1999)	[19]	Switzerland	CVD–ischemic events	310	n/a	* 69	n/a	* 38	* 31	n/a	n/a	20	51	56 ± 16	208	102
Bar et al. (2010)	[20]	Czech Republic	CVD–AIS	45	31	31	n/a	9	0	n/a	n/a	22	22	64.5 ± 13.8 (18–80)	17	14
Lange et al. (2015)	[23]	Brazil	CVD–AIS (21), TIA(3)	265	65	24	n/a	n/a	* 6	n/a	n/a	* 5	* 11	59.25 ± 14.7	14	10
Nasr et al. (2013)	[21]	France	CVD–TIA, AIS	159	116	116	n/a	110	n/a	n/a	n/a	n/a	6	63.4 ± 16.2	86	73
Schlachetzki et al. (2012)	[22]	Germany	CVD–AIS	113	102	86	n/a	76	0	n/a	n/a	10	10	80.6 ± 13.52	50	63
Zunker et al. (2002)	[24]	Germany	CVD	687	29	29	n/a	14	15	n/a	n/a	n/a	15	64 ± 9	419	268
Yang et al. (2018)	[25]	China	CVD–(TIA = 298; Stroke = 105)	403	403	403	93	100	210	92	118	0	210	62.7 ± 8.8	327	76
Tateishi et al. (2008)	[26]	Japan	CVD–(IS = 82, TIA = 7, ICH = 14	120	120	111	n/a	n/a	9	n/a	n/a	3	12	65.5 ± 12.3	69	42
Valaikiene et al. (2008)	[27]	Germany	CVD–IS	40	40	40	* 59	* 5	* 8	* 1	* 7	* 4	* 12	58.9 ± 13.8	26	14
Kunz et al. (2006)	[28]	Germany	CVD	132	132	** 164	** 24	n/a	n/a	n/a	n/a	n/a	** 140	58 ± 14	92	40
Herzberg et al. (2014)	[29]	Germany	CVD–(IS = 73, mimics = 29	232	102	86	76	n/a	n/a	n/a	n/a	10	10	76.8 ± 13.4	48	54
Tang et al. (2005)	[30]	Taiwan	CVD–AIS	193	*** 309	*** 304	n/a	n/a	*** 304	n/a	n/a	0	*** 304	58.3 ± 13.6	158	35
Ogata et al. (2004)	[31]	Japan	CVD—AIS	66	66	55	37	n/a	n/a	n/a	n/a	18	18	63.8 ± 13.1	46	9
Ogata et al. (2005	[38]	Japan	CVD–AIS	75	75	75	44	9	0	n/a	n/a	22	31	64.9 ± 13.1	61	14
Liu et al. (2021)	[41]	China	CVD (AIS, TIA)	1471	375	*** 409	*** 174	*** 103	*** 132	*** 70	*** 62	0	*** 132	62.4 ± 9	318 (88.5	47 (12.5)
Liu et al. (2023)	[39]	China	CVD (MCA stenosis)	104	104	** 208	** 134	** 31	** 43	** 29	** 14	0	** 43	n/a (32–81)	55 (53)	49 (47)
Gerriets et al. (2002)	[40]	Germany and Switzerland	CVD–AIS	58	32	32	18	n/a	n/a	n/a	n/a	10	3	^b^ 64 (38–89)	36 (62)	22 (38)
Lien et al. (2001)	[32]	Taiwan	CVD–AIS	120	120	*** 240	*** 72	n/a	n/a	n/a	n/a	*** 10	n/a	65.1 ± 11.9	62 (52)	58 (48)

CVD—cerebrovascular disease, AIS—acute ischemic stroke symptoms (<24 h); IS—ischemic stroke, TIA—transient ischemic attack; mean age ± SD—mean age ± standard deviation, with ranges in parentheses; ^b^ median age, with interquartile range (IQR) in parentheses; n ( )—number of patients undergoing both index and reference tests, * n—number of stenosis/occlusions identified in vessels, ** n—number of datasets, *** n—number of vessels, n/a—not reported; steno-occlusion—≥50% stenosis + occlusion.

**Table 2:** Main study design characteristics of the included studies. The data extracted are shown in this table: (a) study design, (b) type of ultrasound machine and contrast media, (c) TCCD technique, (d), reference method, (e) time of index to reference test.

Author (s), Publication Year	Ref.	Study Design	Type of Ultrasound Machine and Contrast Media	Ultrasound Technique	Reference Standard	Index to Reference Test Mean Time ± SD
Baumgartner et al. (1999)	[19]	Prospective, single center, consecutive patients	Acuson 128 XP/10 equipped with a 2.0/2.5-MHz 90° sector probe	TCCD	DSA	2 days (0–6)
Bar et al. (2010)	[20]	Prospective, single center, consecutive patients	Philips HDI 5000 (ATL, Bothel, WA, USA) equipped with a phased array 2–4-MHz transducer	TCCD	CTA	12 ± 7.2 (11–20) min
Nasr et al. (2013)	[21]	Retrospective, consecutive	Philips IU 22 (PhilipsUltrasound, Bothell, WA, USA).	TCCD	MRA	4 h
Schlachetzki et al. (2012)	[22]	Prospective, single center, consecutive patients	1. Sonosite Micromaxx equipped with a p17 transducer (sonosite Incl., Bothell, Washington, DC, USA) 2. Philips CX50 with a P2–5 transducer (Philips Ultrasound Bothwell, Wash, USA),	1. ncTCCD (72) and 2. CEUS ncTCCD (41)	MRA, CTA	12 ± 7.2 (11–20) min
Lange et al. (2015)	[23]	Retrospective, single center, non-consecutive	A portable vascular duplex ultrasound (Vivid E1, GE) equipped with a (1.5–5) MHz phased-array probe	ncTCCD	DSA	2 ± 1 days
Zunker et al. (2002)	[24]	Prospective, single center, consecutive patients	HDI 3000 device (ATL). 2–3 MHz phased-array transducer SH U 508A (Levovist, Schering, Berlin, Germany)	CEUS TCCD	MRA	<90 days
Yang et al. (2018)	[25]	Retrospective, single center, non-consecutive	Philips IU22 (Koninklijke Philips N.V., Amsterdam, The Netherlands) and Hitachi Ascendus (Hitachi, Tokyo, Japan) ultrasound systems with 1.0–5.0 MHz phased-array probes	TCCD	DSA	<2 weeks
Tateishi et al. (2008)	[26]	Prospective, single center, consecutive patients	HDI 5000 (Philips, Tokyo, Japan) equipped with a phased-array 2–3 MHz transducer C.A—Levovist, Schering, Berlin, Germany	cTCCD + CEUS cTCCD	DSA	<48 h
Valaikiene et al. (2008)	[27]	Prospective, single center	(Elegra, 2.5PL20, 7.5L40; Siemens, Issaquah, Wash; or Logiq 500, 2.9/2.0S222, 6.7/5.0L739; GE Healthcare, Tokyo, Japan) (2–3 MHz) phased-array transducer	TCCD-Dist ICA	DSA	5 ± 4 days, (0–14) days
Kunz et al. (2006)	[28]	Prospective, single center	Acuson 128XP/4 (Siemens, Berlin and Munich, Germany) equipped with a 2.0/2.5-MHz phased-array transducer, C.A—Levovist, Schering, Berlin, Germany	TCCD + CEUS TCCD	DSA	3 ± 3 days (median, 1.5 days)
Herzberg et al. (2014)	[29]	Prospective, single center, consecutive patients	1. Sonosite Micromaxx equipped with a p17 transducer (sonosite Incl., Bothell, WA, USA) 2. Philips CX50 with a P2–5 transducer (Philips Ultrasound Bothwell, Washington, DC, USA) C.A—UCA; SonoVue, Bracco Imaging SpA, Milan, Italy	TCCD + CEUS TCCD	CTA, MRA	n/a
Tang et al. (2005)	[30]	Prospective, single center, consecutive patients	Philips 4500 (Philips Medical Systems, Bothell, WA, USA) equipped with a 2.0 MHz transducer	cTCCD	MRA	<7 days
Ogata et al. (2004)	[31]	Prospective, single center, consecutive patients	Sonos 5500; Philips Medical Systems, Japan, Tokyo equipped with a 1.0–3.0 MHz phased-array transducer	cTCCD	DSA	24 h
Ogata et al. (2005)	[38]	Prospective, single center, consecutive patients	Sonos 5500; Philips Medical Systems, Japan, Tokyo equipped with a 1.0 –3.0 MHz phased-array transducer	CEUS TCCD	DSA	23.7 h
Liu et al. (2021)	[41]	Retrospective, single center, consecutive patients	Epiq 5 (Philips Medical systems, Amsterdam, The Netherlands) and Hitachi Ascendus (Hitachi, Tokyo, Japan) equipped with a 1–5 MHz phased-array transducer	TCCD	DSA	<2 weeks
Liu et al. (2023)	[39]	Prospective, single center, consecutive patients	PHILIPS EPIQ 7 (C) (Philips, Amsterdam, The Netherlands) equipped with a 1–5 MHz phased-array transducer C.A—SonoVue (Bracco, Milan, Italy)	CEUS TCCD	DSA	n/a
Gerriets et al. (2002)	[40]	Prospective, multi center, consecutive patients	HP Sonos 2000, 2500, or 5500; Acuson 128 XP/10; Toshiba SSH-140 HGor SSH-380; Siemens Elegra. C.A—Levovist, Schering, Berlin, Germany	TCCD + CEUS cTCCD	DSA (1), MRA (18), CTA (13)	<24
Lien et al. (2001)	[32]	Prospective, single center, consecutive patients	HP 5500 equipped with a 2 MHz phased-array transducer	cTCCD	MRA	24 h

ncTCCD—transcranial color-coded Doppler ultrasound (no angle correction, no microbubble contrast), cTCCD—transcranial color-coded Doppler ultrasound (with angle correction, no microbubble contrast), CEUS ncTCCD—transcranial color-coded Doppler ultrasound (no angle correction, with microbubble contrast), CEUS cTCCD—transcranial color-coded Doppler ultrasound (with angle correction, with microbubble contrast), CTA—computed tomography angiography, MRA—magnetic resonance angiography, DSA—digital subtraction angiography, Dist ICA—distal internal carotid artery, n/a—not available.

**Table 3:** Diagnostic performance indicators of transcranial color-coded Doppler ultrasound (TCCD) for the detection of steno-occlusive disease in each of the 30 individual analyses.

Author (s), Publication Year	Ref.	TCCD Technique	Comparator	Site of Stenosis/Occlusion	Degree of Stenosis	Ultrasound Diagnostic Criteria	TTW Failure n or * n (%)	Sen (%)	Spec (%)	PPV (%)	NPV (%)	DA (%)
Baumgartner et al. (1999)	[19]	TCCD	DSA	Ant + Post circulation	≥50%	PSV ≥ 220 MCA	* 280/2741 (10)	100	97.4	100	100	97.4
Baumgartner et al. (1999)	[19]	TCCD	DSA	Ant + Post circulation	<50%	PSV ≥ 155 MCA	* 280/2741 (10)	92.1	90.3	92.1	93	91.3
Bar et al. (2010)	[20]	ncTCCD + TCCD-UCA	CTA	Ant + Post circulation	Occlusion	1. PSV > 220 cm/s 2. absent flow signal + ant arteries visible	5/45 (11)	100	77.8	91.7	100	93.5
Bar et al. (2010)	[20]	* TCCD	CTA	MCA main sterm	Occlusion	MCA flow signal absent + ant arteries visible	5/45 (11)	92.3	94.4	92.3	94.4	93.5
Nasr et al. (2013)	[21]	ncTCCD	MRA	Ant + Post circulation MCA M1 (3), PCA P1 (1), VA (1), BA (1)	≥50% stenosis (5) or occlusion (1)	1.MCA M1 PSV = 220 cm/s, 2. ACA PSV = 155 cm/s 3. PCA P1 PSV = 145 cm/s, 4. BA PSV = 140 cm/s	17/159 (10.7)	100	100	100	100	100
Schlachetzki et al. (2012)	[22]	ncTCCD (72) + CEUS TCCD (41)	CTA, MRA	MCA	Occlusion	MFV-asymmetric index > 21%, absent flow	11/113 (10)	90	98	90	98	98
Lange et al. (2015)	[23]	ncTCCD	DSA	Ant +Post circulation	≥50−occlusion	Retrograde flow, vessel occlusion signals, or turbulent flow patterns	11/65 (17)	72.7	100	100	98.8	98.9
Zunker et al. (2002)	[24]	CEUS TCCD	MRA	Ant +Post circulation	≥50−occlusion	1. MFV > 80 cm/s (MCA and ICA), 2. MFV > 75 cm/s—ACA), 3. MFV > 60 cm/s (PCA and VA), 4. MFV > 65 BA	61 (8.8)	83	82	83.3	82	83
Yang et al. (2018)	[25]	cTCCD	DSA	BA	<50%	PSV ≥ 110	n/a	90.3	80.6	94	71.4	88.1
Yang et al. (2018)	[25]	cTCCD	DSA	BA	50−69%	PSV ≥ 150	n/a	94.3	97.9	98	94	96
Yang et al. (2018)	[25]	cTCCD	DSA	BA	70−99%	PSV ≥ 210	n/a	77.1	98.2	94.5	91.2	92
Yang et al. (2018)	[25]	cTCCD	DSA	BA	<50%	MFV ≥ 70	n/a	87.4	87.1	95.8	67.5	87.3
Yang et al. (2018)	[25]	cTCCD	DSA	BA	50−69%	MFV ≥ 90	n/a	93.8	95.9	96.1	93.4	94.8
Yang et al. (2018)	[25]	cTCCD	DSA	BA	70−99%	MFV ≥ 120	n/a	92.4	90.9	80.7	96.6	91.3
Tateishi et al. (2008)	[26]	CEUS TCCD	DSA	BA	>50%	CEUS PSV > 120 cm/s	6/120 (5)	100	95	64	100	95
Valaikiene et al. (2008)	[27]	TCCD	DSA	Terminal ICA	≥70%	PSV > 200 cm/s	n/a	71	100	100	95.8	96
Kunz et al. (2006)	[28]	CEUS TCCD	DSA	Ant +Post circulation	>0%	increased MFV, retrograde flow, no signals, or turbulent flow patterns.	7/164 (4)	82	98	99	75	84
Herzberg et al. (2014)	[29]	CEUS ncTCCD	CTA, MRA	MCA	MCA occlusion	MCA flow signal absent + ant arteries visible	11/102 (11)	90	98	90	98	97
Tang et al. (2005)	[30]	TCCD	MRA	MCA	>50—near occlusion	PSV ≥ 140 cm/s or PSV < 40 cm/s	17/193 (8.8)	81.8	92.1	48.6	98.2	65.2
Ogata et al. (2004)	[31]	cTCCD	DSA	MCA	MCA occlusion	EDV = 25 cm/s	n/a	94		81	98	94
Ogata et al. (2005)	[38]	CEUS cTCCD	DSA	MCA	>50—MCA stenosis	PSV = 170 cm/s	n/a	89	100	100	99	99.1
Liu et al. (2021)	[41]	cTCCD	DSA	MCA	50−69%	PSV ≥ 180 cm/s	553/1471 (37)	95.7	64	35.6	98.6	69.7
Liu et al. (2021)	[41]	cTCCD	DSA	MCA	70−99%	PSV ≥ 240 cm/s	553/1471 (37)	93.5	89.9	50.7	97.7	85.5
Liu et al. (2021)	[41]	cTCCD	DSA	MCA	50−69%	MFV ≥ 110 cm/s	553/1471 (37)	95.7	64	35.4	98.6	69.4
Liu et al. (2021)	[41]	cTCCD	DSA	MCA	70−99%	MFV ≥ 160 cm/s	553/1471 (37)	91.9	92.8	69.7	97.3	92.2
Liu et al. (2023)	[39]	CEUS * TCCD	DSA	MCA	>0%	PSV ≥ 168.5 cm/s	0/208 (0)	89.2	94.7	39.3	80	97.7
Liu et al. (2023)	[39]	CEUS * TCCD	DSA	MCA	>0%	MFV ≥ 110.5 cm/s	0/208 (0)	90.5	94.7	90.5	94.7	97.5
Gerriets et al. (2002)	[40]	CEUS cTCCD	DSA, MRA, CTA	MCA	>0%	MFV > 120 cm/s, >21% side to side difference	4/58 (7)—CEUS TCCD 32/58 (45)—TCCD	n/a	n/a	n/a	n/a	97
Lien et al. (2001)	[32]	cTCCD	MRA	MCA	>0%	PSV ≥ 120 cm/s	* 89/240 (37)	66.7	90.5	93.9	55.1	78.6
Lien et al. (2001)	[32]	cTCCD	MRA	MCA	>0%	MFV ≥ 85 cm/s	* 89/240 (37)	59.9	92.9	94.8	50.6	76
Lien et al. (2001)	[32]	cTCCD	MRA	MCA	>0%	EDV ≥ 85 cm/s	* 89/240 (37)	52.7	90.5	92.5	46.3	71.6

ncTCCD—transcranial color-coded Doppler ultrasound (no microbubble contrast); cTCCD—transcranial color-coded Doppler ultrasound (angle correction and no microbubble contrast), cTCCD—transcranial color-coded Doppler ultrasound (angle correction and no microbubble contrast); ncTCCD-UCA—transcranial color-coded Doppler ultrasound (no angle correction, with contrast); cTCCD-UCA—transcranial color-coded Doppler ultrasound (with angle correction and contrast), * TCCD—not clear whether angle correction was used; CTA—computed angiography, PMD-TCD—power motion mode transcranial Doppler ultrasound, MRA—magnetic resonance angiography, DSA-digital subtraction angiography, PSV-Peak systolic velocity (cm/s); S-N MFV ratio-stenotic to normal mean flow velocity ratio; MFV asymmetric index—mean flow velocity asymmetry index (ipsilateral and contralateral); na—not available; n—number of patients; * n—number of vessels. Sen—sensitivity; Spec—specificity; PPV—positive predictive value; NPV—negative predictive value; DA—diagnostic accuracy; TP—true positive; TN—true negative; FP—false positive; FN—false negative were calculated based on the given data on sensitivity, specificity, and the number of patients if not given [42]. *—indicates that the value is a calculated value; >0—distinguishing normal from any stenosis. MCA—middle cerebral artery; Ant + Post circulation—anterior and posterior circulation evaluated; BA—basilar artery, Dist ICA—distal internal carotid artery. ncTCCD—transcranial color-coded Doppler ultrasound (no angle correction, no microbubble contrast); cTCCD—transcranial color-coded Doppler ultrasound (with angle correction, no microbubble contrast); ncTCCD-UCA—transcranial color-coded Doppler ultrasound (no angle correction, with microbubble contrast); cTCCD-UCA—transcranial color-coded Doppler ultrasound (with angle correction, with microbubble contrast); CEUS—contrast-enhanced ultrasound; * TCCD—not clear whether angle correction was used; CTA—computed tomography angiography, MRA—magnetic resonance angiography, DSA-digital sub-traction angiography, PSV-Peak systolic velocity (cm/s); MFV—mean flow velocity (cm/s); MFV asymmetric index—mean flow velocity asymmetry index (ipsi-lateral and contralateral); n/a—not available; n—number of patients; * n—number of vessels. Sen—sensitivity; Spec—specificity; PPV—positive predictive value; NPV—negative predictive value; DA—diagnostic accuracy [42]; TTW—transtemporal window; >0—distinguishing normal from any stenosis. MCA—middle cerebral artery; Ant + Post circulation—anterior and posterior circulation evaluated; ACA—anterior cerebral anterior; ICA—internal carotid artery; PCA—posterior cerebral artery; VA—vertebral artery; BA—basilar artery.

**Table 4:** QUADAS 2 methodological quality assessment results (risk of bias and applicability concerns).

Study Author, Date	Ref.	Risk of Bias				Applicability Concerns		
		Patient Selection	Index Test	Reference Standard	Flow and Timing	Patient Selection	Index Test	Reference Standard
Baumgartner et al. (1999)	[19]	Low	Low	Low	Low	Low	Low	Low
Bar et al. (2010)	[20]	Low	Low	Low	Low	Low	Low	Low
Nasr et al. (2013)	[21]	High	Low	Low	Low	Low	Low	Low
Schlachetzki et al. (2012)	[22]	Low	Low	High	Low	Low	Low	High
Lange et al. (2015)	[23]	High	High	Low	Low	High	High	Low
Zunker et al. (2002)	[24]	High	Low	Low	Low	High	Low	Low
Yang et al. (2018)	[25]	High	Low	Low	Low	High	Low	Low
Tateishi et al. (2008)	[26]	Low	Low	Low	Low	Low	Low	Low
Valaikiene et al. (2008)	[27]	Low	Low	Low	Unclear	Low	Low	Low
Kunz et al. (2006)	[28]	Low	Low	Low	Low	Low	High	Low
Herzberg et al. (2014)	[29]	Low	Low	High	Low	Low	Low	High
Tang et al. (2005)	[30]	Low	Low	Low	Low	Low	Low	Low
Ogata et al. (2004)	[31]	Low	Low	Low	Low	Low	Low	Low
Ogata et al. (2005	[38]	Low	Low	Low	Low	Low	Low	Low
Liu et al. (2021)	[41]	Low	Low	Low	Low	Low	Low	Low
Liu et al. (2023)	[39]	Low	High	Low	Low	Low	Low	low
Gerriets et al. (2002)	[40]	Low	High	High	Low	Low	Low	High
Lien et al. (2001)	[32]	Low	Low	Low	Low	Low	Low	Low

**Table 5:** Summary of pooled diagnostic performance of TCCD in stratifying ICAs according to various categories.

Category	Sensitivity (%)—95% CI	Specificity (%)—95% CI	AUC	DOR—95% CI
(All angiographies—DSA, MRA, CTA) as reference standards	83 (81–85)	87 (86–88)	0.96	98 (56–169)
DSA alone as reference standard	90 (88–91)	86 (85–87)	0.97	121 (61–169)
Stratifying stenosis ≥50% to near occlusion (all angiographies)	91 (89–93)	88 (87–89)	0.97	148 (84–262)
Total occlusions	92 (84–97)	98 (96–99)	0.98	148 (84–262)
PSV as diagnostic parameter	85 (82–87)	85 (84–87)	0.96	106 (39–288)
MFV as diagnostic parameter	84 (81–87)	87 (85–88)	0.96	79 (39–157)
non-contrast TCCD	82 (80–84)	88 (87–89)	0.98	94 (56–160)
contrast-enhanced TCCD	87 (83–91)	80 (76–83%)	0.95	87 (13–584)

DSA—digital subtraction angiography, CTA—computed tomography angiography, MRA—magnetic resonance angiography; PSV—peak systolic velocity; MFV—mean flow velocity, TCCD—transcranial color-coded Doppler ultrasound technique.

## Data Availability

The datasets of this study are presented in the article/Supplementary Materials. Any further inquiries can be directed to the corresponding author/s.

## Supplementary Materials

The following supporting information can be downloaded at: https://www.mdpi.com/article/10.3390/jcm13051507/s1, Table S1: Database search strings.
