# Supplementary material for: The Diagnostic Accuracy of Transcranial Color-Coded Doppler Ultrasound Technique in Stratifying Intracranial Cerebral Artery Stenoses in Cerebrovascular Disease Patients: A Systematic Review and Meta-Analysis
Source: J Clin Med. 2024 Mar 5;13(5):1507. doi: 10.3390/jcm13051507 (PMC10934108; doi:10.3390/jcm13051507)
Supplement: Supplementary file 1 [file jcm-13-01507-s001.zip › jcm-2819945-supplementary.pdf]

Table S1: Database search strings.

Searched DataBase	Search strings
Pubmed	(((Cerebrovascular disease) AND (ultrasonography)) AND ((digital subtraction angiography OR computed tomography angiography OR magnetic resonance angiography OR histopathology))) AND ((diagnostic accuracy))
EBSCOhost (CINAHL complete)	TX cerebrovascular disease AND TX ultrasonography AND TX diagnostic accuracy AND TX (digital subtraction angiography OR computed tomography angiography OR magnetic resonance imaging OR histopathology)
Medline	cerebrovascular disease AND ultrasonography AND (digital subtraction angiography OR computed tomography angiography OR magnetic resonance angiography OR histopathology) AND diagnostic accuracy
Embase	('cerebrovascular disease'/exp OR 'cerebrovascular disease') AND ('echography'/exp OR 'echography') AND ('diagnostic accuracy'/exp OR 'diagnostic accuracy')
Web of Science Core Collection	1.ALL=(cerebrovascular disease AND ultrasonography AND (digital subtraction angiography OR computed tomography angiography OR magnetic resonance angiography OR histopathology) AND (diagnostic accuracy))<br>2.ALL=(cerebrovascular disease AND (contrast enhanced ultrasound)) AND (diagnostic accuracy))
Google scholar	cerebrovascular disease AND ultrasonography AND diagnostic accuracy
